# The Effect of Mini-PEG-Based Spacer Length on Binding and Pharmacokinetic Properties of a ^68^Ga-Labeled NOTA-Conjugated Antagonistic Analog of Bombesin

**DOI:** 10.3390/molecules190710455

**Published:** 2014-07-17

**Authors:** Zohreh Varasteh, Ulrika Rosenström, Irina Velikyan, Bogdan Mitran, Mohamed Altai, Hadis Honarvar, Maria Rosestedt, Gunnar Lindeberg, Jens Sörensen, Mats Larhed, Vladimir Tolmachev, Anna Orlova

**Affiliations:** 1 Preclinical PET Platform, Department of Medicinal Chemistry, Faculty of Pharmacy, Uppsala University, Uppsala SE-751 23, Sweden; 2 Biomedical Radiation Sciences, Department of Radiology, Oncology and Radiation Sciences, Faculty of Medicine, Uppsala University, Uppsala SE-751 85, Sweden; 3 Organic Pharmaceutical Chemistry, Department of Medicinal Chemistry, Faculty of Pharmacy, Uppsala University, Uppsala SE-751 23, Sweden; 4 PET Centre, Centre for Medical Imaging, Uppsala University Hospital, Uppsala SE-751 85, Sweden; 5 Department of Medicinal Chemistry, Science for Life Laboratory, BMC, Uppsala University, Uppsala SE-751 23, Sweden

**Keywords:** bombesin analog, PEG, GRPR, antagonist, molecular imaging, breast cancer, prostate cancer, BT-474, PC-3 cells

## Abstract

The overexpression of gastrin-releasing peptide receptor (GRPR) in cancer can be used for peptide-receptor mediated radionuclide imaging and therapy. We have previously shown that an antagonist analog of bombesin RM26 conjugated to 1,4,7-triazacyclononane-*N*,*N*',*N*''-triacetic acid (NOTA) via a diethyleneglycol (PEG_2_) spacer (NOTA-PEG_2_-RM26) and labeled with ^68^Ga can be used for imaging of GRPR-expressing tumors. In this study, we evaluated if a variation of mini-PEG spacer length can be used for optimization of targeting properties of the NOTA-conjugated RM26. A series of analogs with different PEG-length (*n* = 2, 3, 4, 6) was synthesized, radiolabeled and evaluated *in vitro* and *in vivo*. The IC_50_ values of ^nat^Ga-NOTA-PEG*_n_*-RM26 (*n* = 2, 3, 4, 6) were 3.1 ± 0.2, 3.9 ± 0.3, 5.4 ± 0.4 and 5.8 ± 0.3 nM, respectively. In normal mice all conjugates demonstrated similar biodistribution pattern, however ^68^Ga-NOTA-PEG_3_-RM26 showed lower liver uptake. Biodistribution of ^68^Ga-NOTA-PEG_3_-RM26 was evaluated in nude mice bearing PC-3 (prostate cancer) and BT-474 (breast cancer) xenografts. High uptake in tumors (4.6 ± 0.6%ID/g and 2.8 ± 0.4%ID/g for PC-3 and BT-474 xenografts, respectively) and high tumor-to-background ratios (tumor/blood of 44 ± 12 and 42 ± 5 for PC-3 and BT-474 xenografts, respectively) were found already at 2 h p.i. of ^68^Ga-NOTA-PEG_3_-RM26. Results of this study suggest that variation in the length of the PEG spacer can be used for optimization of targeting properties of peptide-chelator conjugates. However, the influence of the mini-PEG length on biodistribution is minor when di-, tri-, tetra- and hexaethylene glycol are compared.

## 1. Introduction

Gastrin-releasing peptide receptor (GRPR) is a cell membrane receptor expressed in almost all the prostate cancer (PC) primary tumors and, frequently, in metastases [1,2]. The receptor expression was observed in 85.7% of lymph node metastases and 52.9% of bone metastases of PC patients [1]. Since normal prostate tissue as well as benign prostate tumors are mostly GRPR-negative, this receptor emerged as an attractive target for new diagnostic approaches and radionuclide therapeutic applications for PC patients [2]. 

The presence of GRPRs has also been documented in breast cancer (BC) [3]. *In vitro* receptor autoradiography on tissue sections has shown that 62% of the primary breast tumors are expressing GRPRs and when the receptor-positive breast cancers spread to local lymph nodes, the expression of GRPRs has been kept in the metastatic deposit [4]. It has been shown that all the metastases of the receptor-positive primary tumors are GRPR-positive and the expression density of primary tumors and metastases are generally high [4]. It has been also shown that these receptors contribute to the metastatic process by increasing cellular migration in BC [5]. The prevalence of these receptors in BC has led to develop GRPR-targeted diagnostic and therapeutic peptide-based pharmaceuticals [6,7].

Bombesin (BN), a linear amphibian tetradecapeptide, is an analog of the mammalian gastrin-releasing peptide (GRP) and binds to GRPRs with high affinity and selectivity. Because of the poor *in vivo* stability of GRP, appreciable efforts have been made in the development of BN analogs for targeting of GRPR [8]. Different BN analogues were labeled with cytotoxic beta- and alpha-particle emitting nuclides ^90^Y [9], ^177^Lu [9,10] and ^212/213^Bi [11] for radionuclide therapy, and with gamma- and positron-emitting radionuclides ^111^In [9], ^99m^Tc[12,13,14], ^68^Ga [13] and ^64^Cu [13,15] for visualization of GRPR-expressing tumors. BN shows high structural and functional homology with GRP. They share seven amino acids amidated C-terminus sequence homology, Trp*-*Ala-Val-Gly-His-Leu-Met-NH_2_. It has been shown that these C-terminal seven amino acids are responsible for binding to the GRPRs and any modification near C-terminus of BN analogs decreases the binding affinity [16]. Therefore, the N-terminus of these analogs was coupled to the chelators for loading with radiometals or modified for labeling with other radionuclides [13,15].

It has been shown that direct coupling of the radiometal-chelator complex to the N-terminal of the BN analogs decreases the receptor-binding affinity [17], while the application of a spacer prevents the interference of the radiometal-chelator complex with the binding site of the targeting vector to the GRPR [18]. The analog containing no spacer (X = 0) for ^111^In-DOTA-X-BN[7,8,9,10,11,12,13,14]NH_2_ has exhibited 100-fold lower binding affinity to GRPR in comparison with a 8-carbon aliphatic spacer containing analog [18]. Lys(sha)-βAla-βAla spacer for coupling of retro[N^α^-carboxymethyl-histidine] to BN analogs for ^99m^Tc(CO)_3_ labeling improved the affinity of conjugates by factor 23 [12]. It has also been demonstrated that excessive increase of the lipophilicity of the linker reduces the affinity [18]. Significantly higher liver uptake for hydrophobic spacer-containing analogs adds to the problem of the suboptimal pharmacokinetics of these analogs [18]. To improve the pharmacokinetic profiles for BN analogs, more hydrophilic spacer moieties were introduced. Schweinsberg *et al.* have shown that hydrophilic carbohydrate groups introduced into the linker sequence of ^99m^Tc-labeled BN analogs decreased liver uptake significantly [19]. Increasing the charge of the spacer by insertion of negatively charged β^3^hGlu, demonstrated favorable biodistribution for a BN analog labeled using ^99m^Tc-tricarbonyl core [20].

The polyethylene glycol (PEG) has been widely used for modification of therapeutic peptides and proteins [21]. PEGylation, the covalent attachment of the PEG to the biologically active molecule, is a modification methodology with the main purpose of reducing immunogenicity and rapid enzymatic degradation of the proteins [22]. The PEG_4_ spacer has been introduced in BN agonist (DOTA-PESIN) with the aim to increase its metabolic stability and to improve its tumour accumulation [23]. This has been found to be successful, moreover, an appreciable switch of elimination pathway from hepatic to renal was observed. PEGylation can also increase the overall hydrophilicity of the modified peptides and proteins [24,25]. However, introduction of PEG_3_ spacer into fluorinated RGD-based integrin-targeted peptide did not affected its hepatic uptake [26].

PEGylation might improve the pharmacokinetic properties of targeting molecules by appreciable reduction of the hepatic uptake and/or hepatobiliary excretion of the radiotracers. For example, a replacing the lipophilic 6-aminocaporoic acid (CA) linker with more hydrophilic PEG_4_ linker could enhance the clearance kinetics of ^99m^Tc-labeled cyclic RGD peptide with minor impact on binding affinity to GPIIb/IIIa receptors [27]. The PEGylated BN(7–14) analog, where PEG with the length of 5–20 kDa was conjugated to the BN radiolabeled using ^99m^Tc(CO)_3_, exhibited higher *in vitro* and *in vivo* stability [14]. It has also been shown that PEGylation did not affect the binding affinity although slower *in vitro* kinetics was found for PEG-conjugated BN analogs. Fast blood clearance via renal elimination and decreased hepatobiliary excretion led to higher tumor-to-non-tumor ratios for PEG-modified (5 kDa) BN in comparison with non-PEGylated analogs [14]. 

It should be taken into consideration that small modifications in the bioactive molecular targeting vectors can influence their biodistribution pattern and tumor targeting properties. The number of monomer units of polymer plays significant role in the biological behavior of PEG-modified conjugates. Because of the flexibility of the PEG chain, there is a risk of masking the biologically active receptor recognition site of the peptide and subsequently impairment of binding affinity by using long PEG spacers or chains [28]. There are some examples in the literature where PEGylated proteins exhibited unexpected behavior of increased activity or PEGylation changed the biological effects [29,30]. Asparaginase, an enzyme of therapeutic interest, exhibited increased activity after modification with mPEG_2_-COOSu [29]. It has further been shown that PEGvisomant, the PEGylated form of growth hormone receptor antagonist, showed lower antagonistic function compared with its non-PEGylated core molecule B2036 [30]. Thus, PEG modification can affect the pharmacological activity of drugs and therefore the balance between the degree of PEG modification and the molecular size of the drug should be considered [31].This influence could be even more pronounced for short peptides. Hence, modification in the length of the spacer moiety might influence the BN binding affinity to GRPRs and its *in vivo* kinetics. 

We have previously investigated an antagonist analog of BN (D-Phe-Gln-Trp-Ala-Val-Gly-His-Sta-Leu-NH_2_, RM26) conjugated to 1,4,7-triazacyclononane-*N*,*N*',*N*''-triacetic acid (NOTA) via a diethylene glycol (PEG_2_) spacer (NOTA-PEG_2_-RM26) labeled with ^68^Ga, ^111^In and Al ^18^F [32,33]. This conjugate showed favorable properties for *in vivo* imaging of GRPR-expression in PC. 

In this study we investigated the possibility to modulate the lipophilicity and thus liver uptake of the conjugate by introducing extended mini-PEG linkers. Since the liver is one of the major metastatic sites for BC, high radioactivity accumulation in the liver is an undesirable property for the imaging agents. 

For this purpose, a series of NOTA-PEG*_n_*-RM26 (*n* = 2, 3, 4 and 6) conjugates were synthesized. The peptides were labeled with a short-lived generator-produced radionuclide ^68^Ga (T_½_ = 67.8 min) for imaging using positron emission tomography (PET). *In vitro* and *in vivo* studies were performed using PC-3 (PC cells) and BT-474 (BC cells). More long-lived ^111^In (T_½_ = 2.8 days) was used as a label in some *in vitro* experiments requiring longer time than it is permissible by the short half-life of ^68^Ga.

## 2. Results and Discussion

### 2.1. Peptide Synthesis

HPLC/MS analysis of the final products NOTA-PEG***_n_***-RM26 using Kinetex 2.6 µm C18 (50 × 3.0 mm) column and 2.5 min, 5%–60% acetonitrile/water (0.05% formic acid) gradient gave M/Z values in accordance with those expected for all the variants (Table 1). The purity, as determined by UV-HPLC (220 nm), was over 98% for all the conjugates.

**Table 1:** Characterization of NOTA-PEG*_n_*-RM26, (*n* = 2, 3, 4 and 6).

	PEG_2_	PEG_3_	PEG_4_	PEG_6_
Synthetic yield (%)	24	26	33	26
*m/z* value (*m/z* theor. value)	1543.88 (1542.82)	1601.58 (1600.86)	1646.06 (1644.89)	1734.23 (1732.94)
Purity (%)	98.3	99.3	99.8	99.5

### 2.2. Radiolabeling and Stability Test

The radiolabeling yield for ^68^Ga-and ^111^In-NOTA-PEG*_n_*-RM26 was typically over 98% (Table 2). A maximum specific radioactivity of 28 GBq/µmol and 6 GBq/µmol was obtained for ^68^Ga- and ^111^In-labeled NOTA-PEG*_n_*-RM26, respectively. Because of almost quantitative radiolabeling yields these tracers were used without further purification for *in vitro* studies while ^68^Ga-NOTA-PEG*_n_*-RM26 analogs were purified for *in vivo* studies using cartridge purification. All ^68^Ga- and ^111^In-labeled analogs demonstrated high stability after challenging with 1,000-fold molar excess of EDTA for 1 h at RT (Table 2). All NOTA-conjugated BN analogs were hydrophilic. *LogD* values of the ^68^Ga-labeled peptides were in the range between −2.27 ± 0.07 for ^68^Ga-NOTA-PEG_2_-RM26 and −2.50 ± 0.09 for ^68^Ga-NOTA-PEG_6_-RM26 (Table 2). There was a small but significant increase of the overall hydrophilicity of the conjugates with increasing length of the spacer from 2 to 3 ethyleneglycol units.

**Table 2:** Labeling, stability and octanol-PBS distribution coefficients of NOTA-PEG*_n_*-RM26, (*n* = 2, 3, 4 and 6).

	PEG_2_	PEG_3_	PEG_4_	PEG_6_
Labeling yield for ^68^Ga, %	99.4 ± 0.3	98 ± 1	99.2 ± 0.1	99.3 ± 0.1
Stability under EDTA challenge, %	99.3 ± 0.1	98.1 ± 0.2	98.5 ± 0.1	99.1 ± 0.2
Labeling yield for ^111^In, %	96 ± 2	97 ± 2	97 ± 2	97 ± 2
Stability under EDTA challenge, %	95.5 ± 0.3	92.5 ± 0.7	94.3 ± 0.6	94.6 ± 0.7
*LogD* of ^68^Ga-NOTA-PEG*_n_*-RM26	−2.27 ± 0.07	−2.47 ± 0.06	−2.49 ± 0.10	−2.50 ± 0.09

### 2.3. In Vitro Studies

#### 2.3.1. *In Vitro* Binding Specificity Assay

To ensure that the GRPR-binding capacity of NOTA-PEG*_n_*-RM26 was preserved after labeling, *in vitro* binding specificity tests was performed using PC-3 and, for NOTA-PEG_3_-RM26, BT-474 cells. The binding of ^68^Ga- and ^111^In-labeled NOTA-PEG*_n_*-RM26 to these GRPR-expressing cells was receptor mediated since pre-saturation of the cells with non-labeled peptides significantly decreased the cell binding of the radiolabeled compounds (Table 3).

**Table 3:** Binding specificity and cellular processing of NOTA-PEG*_n_*-RM26, (*n* = 2, 3, 4 and 6).

	PEG_2_	PEG_3_	PEG_4_	PEG_6_
*In vitro* binding specificity test, % blocked uptake of ^68^Ga-labeled variants on PC-3 (BT474) cells in presence of excess of non-labeled tracer	94.5 ± 0.1	98.0 ± 0.1 (95.3 ± 0.2)	98.2 ± 0.1	95.5 ± 0.3
*In vitro* binding specificity test, % blocked uptake of ^111^In-labeled variants on PC-3 (BT474) cells in the presence of excess of non-labeled tracer	97.7 ± 0.2	94.0 ± 0.2 (95.0 ± 0.4)	95.0 ± 0.9	94.8 ± 0.4
Internalization rate by PC-3 (BT474) cells, % internalized radioactivity from total cell associated at 24 h continuous incubation	33 ± 3	31 ± 2 (32 ± 2)	30 ± 1	35 ± 2

#### 2.3.2. Cellular Uptake and Internalization

**Figure 1:**
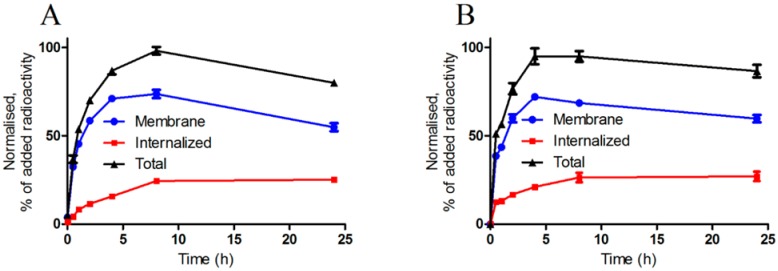
Cell-associated (internalized, membrane-bound and total) radioactivity as a function of time after continuous incubation of (**A**) PC-3 and (**B**) BT-474 cells with ^111^In-NOTA-PEG_3_-RM26. Data are mean values ± SD of 3 culture dishes. Not all error bars are visible due to the small standard deviations.

Figure 1 presents the binding of ^111^In-NOTA-PEG_3_-RM26 to the PC-3 and BT-474 cells. Binding and processing of other ^111^In-NOTA-PEG*_n_*-RM26 conjugates to PC-3 cells was very similar to binding and processing of ^111^In-NOTA-PEG_3_-RM26 (data not shown). The binding was rapid, and the cell-associated radioactivity reached a plateau between 4 h and 8 h. The internalization rate of the ^111^In-labeled conjugates was slow. After 24 h of incubation, the contribution of the internalized radioactivity to the overall cell-bound radioactivity was 30%–35% for both of the cell lines (Table 3).

#### 2.3.3. Real-Time Ligand Binding Kinetics: KD and Bmax Determination

LigandTracer measurements showed that all conjugates had rapid on-rate and slow off-rate (Table 4). The calculated K_D_ values for ^111^In-NOTA-PEG*_n_*-RM26 were in the low picomolar range. The number of binding sites was 417,000 ± 12,000 and 157,000 ± 7,000 receptors/cell for PC-3 and BT-474 cells, respectively, as measured using ^111^In-NOTA-PEG_3_-RM26.

**Table 4:** Kinetics of NOTA-PEG*_n_*-RM26, (*n* = 2, 3, 4 and 6) binding to living PC-3 cells.

	PEG_2_	PEG_3_	PEG_4_	PEG_6_
Association rate, k_a_, M^−1^ s^−1^	(2.9 ± 0.3) × 10^5^	(8 ± 4) × 10^5^	(5 ± 3) × 10^5^	(6 ± 5) × 10^5^
Dissociation rate, k_d_, s^−1^	(11.2 ± 0.0) × 10^−6^	(2.7 ± 0.2) × 10^−6^	(2.5 ± 0.0) × 10^−6^	(7 ± 3) × 10^−6^
K_D_, pM	23 ± 13	5 ± 3	8 ± 5	18 ± 8

**Figure 2:**
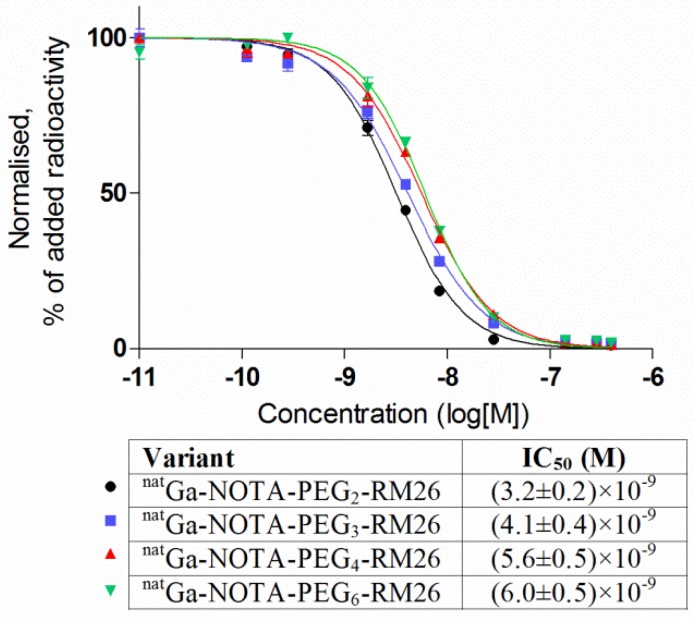
Inhibition of ^125^I-Tyr^4^-BBN binding to PC-3 cells with ^nat^Ga-NOTA-PEG*_n_*-RM26 (*n* = 2, 3, 4 and 6). Data are presented as mean values of three dishes ± SD.

#### 2.3.4. *In Vitro* Competitive Binding Assay: IC_50_ Determination

The binding properties of ^nat^Ga-loaded analogs were compared in a competitive binding assay using ^125^I-Tyr^4^-BBN as the displacement radioligand (Figure 2). The IC_50_ values of ^nat^Ga-NOTA-PEG*_n_*-RM26 increased with the increase of the mini-PEG spacer length. The difference in IC_50_ was within factor two for ^nat^Ga-NOTA-PEG_2_-RM26 and ^nat^Ga-NOTA-PEG_6_-RM26, and there was no difference between valuses for ^nat^Ga-NOTA-PEG_4_-RM26 and ^nat^Ga-NOTA-PEG_6_-RM26.

### 2.4. In Vivo Studies

#### 2.4.1. Biodistribution in NMRI Mice

The biodistribution of ^68^Ga-NOTA-PEG*_n_*-RM26 in female NMRI mice was performed to assess their blood and whole body clearance. Data at 1 and 2 h p.i. are presented in Table 5. All ^68^Ga-labeled analogs showed rapid whole body and blood clearance. Low radioactivity in the GI tract (with content) suggests that the renal excretion was predominant. Low kidney retention and liver uptake was found for all the conjugates. Significantly lower radioactivity accumulation was found in the liver for ^68^Ga-NOTA-PEG_3_-RM26 compared to PEG_4_ and PEG_6_ variants. It should be noted that the decrease of the hepatic uptake was minor, as all uptake values were in the range of 0.6–1.1%ID/g at 1 h p.i. Furthermore, the difference between the hepatic uptake of PEG_2_ and PEG_3_-bearing peptides was not significant (despite their differences in *logD*). Uptake of all tested conjugates in GRPR-expressing organs (pancreas and organs of GI) was high at 1 h p.i., but radioactivity was significantly decreased in these organs already 2 h p.i. 

**Table 5:** Biodistribution of ^68^Ga-NOTA-PEG*_n_*-RM26 (*n* = 2, 3, 4 and 6) in female NMRI mice. Data are presented as the mean percentage of the injected dose per gram of tissue (%ID/g ± SD, *n* = 3).

	PEG_2_		PEG_3_				PEG_4_		PEG_6_	
Organ	1 h	2 h	1 h		2 h		1 h	2 h	1 h	2 h
**Blood**	0.4 ± 0.2	0.05 ± 0.01		0.19 ± 0.02	0.065 ± 0.002	0.25 ± 0.06		0.073 ± 0.007	0.27 ± 0.06	0.06 ± 0.03
**Lung**	0.3 ± 0.1	0.5 ± 0.4		0.20 ± 0.05 ^a^	0.17 ± 0.01 ^a^	0.32 ± 0.06		0.5 ± 0.1	0.5 ± 0.2	0.14 ± 0.08
**Liver**	0.9 ± 0.2	0.8 ± 0.2		0.6 ± 0.1 ^a,b^	0.7 ± 0.1 ^a,b^	1.1 ± 0.2		1.2 ± 0.2	1.06 ± 0.04	1.1 ± 0.1
**Spleen**	0.6 ± 0.2	0.46 ± 0.03		0.40 ± 0.04 ^b^	0.3 ± 0.1 ^a^	0.5 ± 0.2		0.6 ± 0.1	0.7 ± 0.1	0.6 ± 0.2
**Pancreas**	7 ± 3	1.7 ± 0.6		6.6 ± 0.4	1.7 ± 0.2	6.1 ± 0.6		2.3 ± 0.7	6.2 ± 0.7	1.4 ± 0.3
**Stomach**	1.2 ± 0.3	0.63 ± 0.06		1.0 ± 0.2	0.60 ± 0.04	0.90 ± 0.08		0.7 ± 0.1	1.2 ± 0.2	0.57 ± 0.06
**Sm. intest.**	2.1 ± 0.8	0.8 ± 0.4		2.0 ± 0.3	0.9 ± 0.3	2.0 ± 0.3		1.1 ± 0.6	1.1 ± 0.6	0.51 ± 0.09
**Kidney**	3 ± 2	1.0 ± 0.4		1.6 ± 0.3 ^b^	1.0 ± 0.1	1.7 ± 0.6		1.3 ± 0.2	2.7 ± 0.5	1.08 ± 0.09
**Muscle**	0.10 ± 0.05	0.04 ± 0.02		0.06 ± 0.01	0.019 ± 0.002	0.09 ± 0.03		0.03 ± 0.02	0.08 ± 0.03	0.03 ± 0.02
**Bone**	0.18 ± 0.07	0.07 ± 0.02		0.12 ± 0.04	0.050 ± 0.003	0.12 ± 0.05		0.10 ± 0.04	0.13 ± 0.04	0.11 ± 0.06
GI *	4 ± 2	2.88 ± 0.06		3.7 ± 0.8	2.5 ± 0.7	3.5 ± 0.6		3.1 ± 0.9	2.8 ± 0.2	1.8 ± 0.5
Carcass *	4 ± 1	0.9 ± 0.4		2.1 ± 0.4	0.7 ± 0.1	2.3 ± 0.5		1.2 ± 0.3	3 ± 1	0.71 ± 0.05

* Uptakes in GI and carcass are presented as %ID per whole sample; *^a^* Indicates that the ^68^Ga-NOTA-PEG_3_-RM26 uptake was significantly lower than ^68^Ga-NOTA-PEG_4_-RM26 uptake; *^b^* Indicates that the ^68^Ga-NOTA-PEG_3_-RM26 uptake was significantly lower than ^68^Ga-NOTA-PEG_6_-RM26 uptake.

#### 2.4.2. Biodistribution and *in vivo* Binding Specificity Test in Female BALB/c nu/nu Mice Bearing PC-3 and BT-474 Xenografts

The results for *in vivo* binding specificity test for ^68^Ga-NOTA-PEG_3_-RM26 in PC-3 and BT-474 xenografted mice are presented in Table 6. Tumors and receptor-positive normal organs showed GRPR-specific uptake. Pre-saturating the receptors with co-injection of non-labeled peptide decreased the tumor uptake almost 5-fold, from 4.6 ± 0.6%ID/g to 0.97 ± 0.04%ID/g for PC-3 tumors and more than 7-fold, from 2.8 ± 0.4%ID/g to 0.4 ± 0.01%ID/g for BT-474 tumors. The radioactivity uptake in normal organs except in liver and spleen was similar for both xenograft models. Almost 3-fold lower liver and more than 4-fold lower spleen uptake was found for BT-474 xenografted mice compared to PC-3 tumor bearing animals.

**Table 6:** *In vivo* binding specificity test and biodistribution of ^68^Ga-NOTA-PEG_3_-RM26 in female BALB/c nu/nu mice bearing PC-3 or BT-474 xenografts 2 h p.i. The data are presented as %ID/g ± SD, n = 4.

	PC-3 Cells		BT474 Cells	
Organ	Non-Blocked	Blocked	Non-Blocked	Blocked
Blood	0.11 ± 0.02	0.060 ± 0.010 *	0.05 ± 0.02	0.17 ± 0.06
Liver	1.6 ± 0.1	1.6 ± 0.2	0.53 ± 0.06	0.81 ± 0.08
Spleen	1.4 ± 0.3	0.9 ± 0.3	0.3 ± 0.2	0.4 ± 0.1
Pancreas	3.9 ± 0.6	0.27 ± 0.04 *	2.8 ± 0.6	0.3 ± 90.04 ^*^
Small intestine	1.2 ± 0.4	0.21 ± 0.08 *	0.66 ± 0.08	0.48 ± 0.09 ^*^
Kidney	1.7 ± 0.2	3.3 ± 0.2 *	1.65 ± 0.09	4.25 ± 0.05 ^*^
Tumor	4.6 ± 0.6	0.97 ± 0.04 *	2.8 ± 0.4	0.4 ± 0.1 ^*^
Muscle	0.06 ± 0.02	0.030 ± 0.003	0.05 ± 0.02	0.13 ± 0.04
Bone	0.13 ± 0.03	0.13 ± 0.04	0.08 ± 0.03	0.14 ± 0.05

* Indicates that uptake was significantly lower in blocked than non-blocked groups.

**Figure 3:**
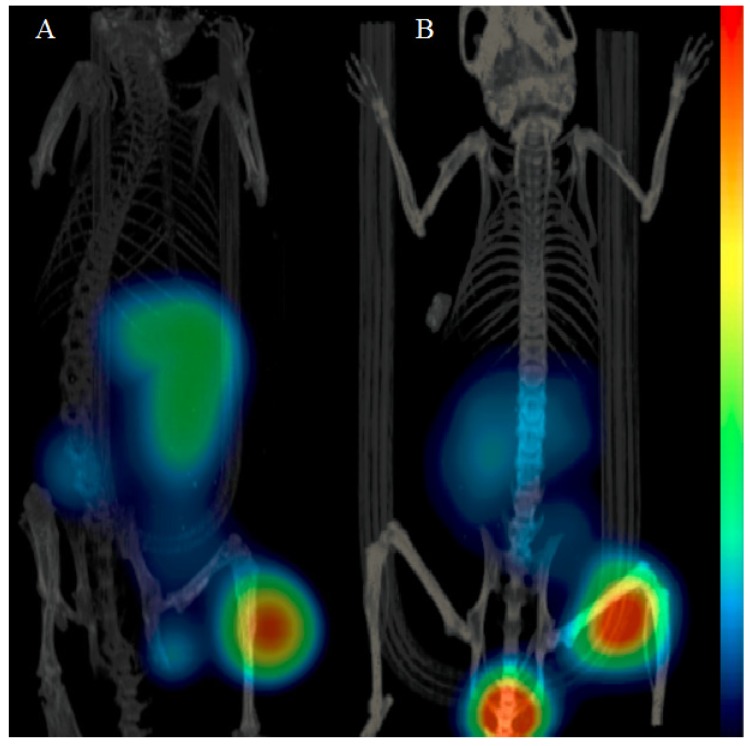
Imaging of GRPR expression 2 h p.i. in (A) PC-3 and (B) BT-474 xenografts in BALB/c nu/nu female mice. The mice were injected with 45 pmol of ^68^Ga-NOTA-PEG_3_-RM26.

### 2.5. Imaging Studies

The mice with PC-3 or BT-474 xenografts injected with ^68^Ga-NOTA-PEG_3_-RM26 were imaged 2 h p.i. (Figure 3). The tumors (right hind legs) were clearly visualized for both models, a finding that was in good agreement with the biodistribution data. Accumulation of radioactivity was also detected in the kidneys and abdominal area.

### 2.6. Discussion

BN-based tracers are promising molecules for radionuclide imaging as well as therapy for selective targeting of GRPR-expressing malignancies, such as prostate and breast tumors. Many of the BN conjugates showed high liver uptake and tendency for predominant hepatobiliary excretion owing to their high lipophilicity [34,35]. Although PC may rarely spread to the liver in very late stage (when imaging is not relevant anymore), this organ is one of the most common sites that BC spreads to. It would be desirable to use the same imaging agent for visualization of both PC and BC. Hence, low unspecific uptake of radiotracers in the liver is essential, as it would provide a high tumor-to-background contrast for BC. Different strategies were taken into consideration in order to tackle the problem of high unspecific liver uptake of BN-analogs [9,36,37,38,39]. It has been demonstrated that introduction of hydrophilic spacers can compensate highly lipophilic character of BN analogs [12,19,20,39]. One strategy for increasing hydrophilicity of BN is the use of mini-PEG as biodistribution modifier [13,14]. Previously, we showed that ^68^Ga-NOTA-PEG_2_-RM26, having PEG_2_ spacer between the chelator and the peptide had favorable biodistribution profile, *i.e.*, rapid blood clearance, low hepatobiliary excretion, low kidney re-absorbtion, high and stable tumor uptake, and rapid washout from normal GRPR-positive organs [32]. As minor modifications in structure of short peptides [40] could lead to substantial effect on their targeting properties, we evaluated if the use of mini-PEG polymer with different length (2, 3, 4 or 6 units) can be used for modification of binding and biodistribution properties of NOTA-PEG*_n_*-RM26 conjugates.

A surprisingly low impact on the biological outcome was observed when different mini-PEG lengths were investigated. This indicates that the preferred length of the mini-PEG chain in this case can be based on straightforwardness of synthesis or purification of the peptides instead of the biodistribution or clearance. In our case, all the peptide analogs discussed were obtain in a similar yield and equally easely purified, but the starting material for the shorter analogs were much easier to handle due to their crystalline appearance compered to PEG_6_ that was an oil.

The increase of mini-PEG length from PEG_2_ to PEG_3_ resulted in a small but significant increase of overall hydrophilicity of ^68^Ga-labeled conjugates (Table 2). Further increase of hydrophilicity was not significant.

It might be expected that the introduction of a longer mini-PEG moiety impairs the affinity of the binding sequence of NOTA-PEG*_n_*-RM26 to the receptors. Indeed, ^111^In-NOTA-PEG_3_-RM26 demonstrated the lowest K_D_ value. However, all of the ^111^In-labeled variants showed the high affinity for GRPR and bound with subpicomolar K_D_ values to the receptors. The IC_50_ values of ^nat^Ga-NOTA-PEG*_n_*-RM26 were also in the same low nanomolar range, and ^68^Ga-NOTA-PEG_3_-RM26 showed one of the lowest half inhibition concentrations. It is notable that IC_50_ and K_D_ values differ in three orders of magnitude for the studied conjugates. It should be mentioned that both these parameters reflect affinity of the conjugate to its receptors. However, accuracy of IC_50_ measurements relies on equilibrium being reached during the time of experiment. This has been identified as a potential source of underestimation of binding capacity, since high affinity binders may have equilibration time of many hours [41]. On the opposite K_D_ values in this study were measured by monitoring the ligand-receptor binding in real-time and did not depend on equilibrium. Moreover, time-resolved interaction measurements provide information about binding kinetics (the association and dissociation rates) [42].

Blood clearance rate via kidney ultrafiltration in NMRI mice was fast for all studied conjugates with no significant difference (Table 5). No significantly different uptake was found in majority of non-target organs, or in receptor-positive organs (Table 5). However, radioactivity accumulations of ^68^Ga-NOTA-PEG_3_-RM26 in the liver was slightly but significantly lower (*p* < 0.05) than uptake of ^68^Ga-NOTA-PEG_4_-RM26 and ^68^Ga-NOTA-PEG_6_-RM26. On the basis of the biodistribution data in NMRI mice and affinity data, we selected ^68^Ga-NOTA-PEG_3_-RM26 as the most suitable conjugate for biodistribution and *in vivo* binding specificity study in tumor bearing mice. In a good agreement with our previous data [32,33], specific uptake was found in receptor-positive abdominal area organs (pancreas as the most abundant receptor expressing organ, stomach and small intestine) and the tumors (Table 6). Both the tumor xenografts were visualized with microPET/CT at 2 h p.i. ^68^Ga-NOTA-PEG_3_-RM26 provided images with well detectable tumors and residual renal radioactivity. Even BT-474 xenografts with lower level of gene expression (almost a third of the expression level in PC-3 cells) were visualized clearly after 2 h p.i. The liver and spleen uptake was significantly lower for BT-474 xenografted mice both in *ex vivo* measurements and in imaging. We speculate that implanted estradiol pellets may affect experimental outcomes.

This study demonstrated the suitability of BN antagonistic analogs, NOTA-PEG*_n_*-RM26 (*n* = 2, 3, 4 and 6), for visualization of GRPR-expression in prostate and breast tumors with favorable excretion kinetics. The hepatic uptake was very low for all the conjugates and the marginal difference in liver uptake for different variants suggests that the addition of only few PEGs to the linker sequence is not sufficient to dramatically change the biodistribution of this peptide. However, it is still suitable for a fine tuning of the targeting properties. The size and lipophilicity of the present analogs were optimized for the imaging of GRPR-expressing tumors with high contrast within a short time after administration both for PC with metastases in the lower abdomen and BC with liver metastases.

## 3. Experimental Section

### 3.1. Peptide Synthesis

**Figure 4:**
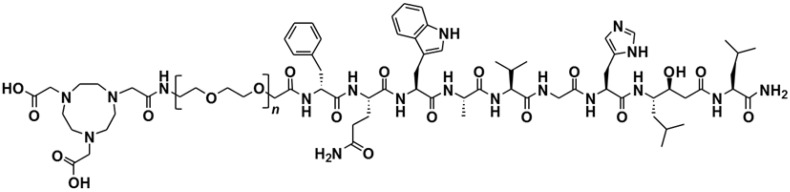
Structure of NOTA-PEG*_n_*-[D-Phe^6^, Sta^13^, Leu^14^]-Bombesin[6,7,8,9,10,11,12,13,14], NOTA-PEG*_n_*-RM26, (*n* = 2, 3, 4 and 6).

NOTA-PEG*_n_*-[D-Phe^6^, Sta^13^, Leu^14^]Bombesin[6,7,8,9,10,11,12,13,14] (*n* = 2, 3, 4 and 6), NOTA-PEG_n_-RM26 (Figure 4) were synthesized by manual solid-phase peptide synthesis (SPPS) using standard Fmoc/*t*-Bu conditions as described earlier [32].

### 3.2. Radiolabeling and Stability Test

The ^111^In-, ^68^Ga- and ^nat^Ga-labeling and quality controls were performed based on protocols presented earlier [32]. Buffers for labeling were purified from metal contamination by Chelex 100 resin (Bio-Rad Laboratories, Hercules, CA). For ^68^Ga labeling, 10 µL (aqueous solution of 10 nmol) of NOTA-PEG*_n_*-RM26 buffered with 250 µL of sodium acetate (Merck, Darmstadt, Germany) to obtain pH 3.9 was incubated with 1 mL ^68^Ga-containing eluate (600 MBq) for 10 min at 75 °C. For cold isotope loading, 14 nmol of ^nat^GaCl_3_, aq. solution (50 µL), (Sigma-Aldrich, St. Louis, Missouri, MO, USA) were added to 10 nmol of NOTA-PEG*_n_*-RM26 using the same protocols as for radioactive isotopes.

The reaction mixtures of ^68^Ga-NOTA-PEG*_n_*-RM26 were purified using solid phase extraction. Briefly, the reaction mixtures were diluted with 3 mL of deionized water and passed through a 1 mL Oasis HLB cartridge (Waters, Milford, MA, USA). The cartridge was then washed with 5 mL of deionized water to remove any unreacted ^68^Ga. The radiolabeled product was eluted with 1 mL of 1:1 EtOH/water. The chemical and radiochemical purity of ^68^Ga-NOTA-PEG*_n_*-RM26 after purification was checked by UV-radio-HPLC. The conditions were as follows: A = 10 mM TFA; B = 70% acetonitrile (MeCN), 30% H_2_O, and 10 mM TFA with UV-detection at 220 nm; gradient elution: 0–2 min at 35% B, 2–9 min at 35 to 100% B, 9–12 min at 100% B; and flow rate was 2.0 mL/min. The analytes were separated using an analytical column with a stationary phase consisting of covalently bonded pentylsilane (Discovery BIO Wide Pore C5; 5 cm × 4.6 mm).

For routine control, radiochemical purity of ^68^Ga- and ^111^In-NOTA-PEG*_n_*-RM26 was analyzed by ITLC (Biodex Medical Systems, Shirley, NY, USA) using 0.2 M citric acid (pH 2.0) as the mobile phase. The method was cross-calibrated with HPLC (see above). In this system free indium, gallium and their complexes migrate with the solvent front (R*f* = 1.0), while peptides remain at the origin (R*f* = 0.0).

For ^111^In labeling, the pH of 10 µL (aqueous solution of 10 nmol) of NOTA-PEG*_n_*-RM26 was adjusted to 5.5 using 80 µL of 0.2 M ammonium acetate (Merck) and mixed with 80–150 µL (~60 MBq) of ^111^In (Covidien, Dublin, Ireland). The mixture was incubated at 80 °C for 30 min.

To evaluate labeling stability, ^68^Ga-NOTA-PEG*_n_*-RM26 or ^111^In -NOTA-PEG*_n_*-RM26 were challenged with 1000-fold molar excess of disodium salt of EDTA (Sigma) and incubated for 1 h at room temperature (RT).

For determination of *logD*, n-octanol (750 µL) was added to a tube with 750 μL of PBS (pH 7.4) containing 0.1 nmol of a ^68^Ga-labeled conjugate, the tube was vigorously vortexed and then centrifuged (14,000 rpm) for 4 min. Aliquots of 50 μL were taken from each phase, and their radioactivity was measured in an automated gamma-counter with 3-inch NaI(Tl) detector (1480 WIZARD, Wallac OyTurku, Finland). Each measurement was repeated nine times. The distribution coefficient was calculated as the average log of a ratio of the radioactivity in the octanol and the PBS fractions.

### 3.3. In Vitro Studies

GRPR expressing human PC cell line PC-3 and human BC cell line BT-474 (ATCC) were cultured in RPMI media complemented with 10% FBS, 2 mM l-glutamine and PEST (penicillin 100 IU/mL) (all from Biochrom AG, Berlin, Germany). This media is referred as complete media in the text. In all *in vitro* experiments, cells were incubated in complete medium and detached using trypsin-EDTA solution (0.25% trypsin, 0.02% EDTA in buffer; Biochrom AG). All experiments were performed in triplicate and 1 × 10^6^ cells/dish were seeded one day before the experiment.

#### 3.3.1. *In Vitro* Binding Specificity Assay

The binding specificity of ^68^Ga- and ^111^In-labeled NOTA-PEG*_n_*-RM26 was tested on PC-3 and BT-474 cells. The cells were incubated with 1 nM concentration of ^68^Ga- and ^111^In-NOTA-PEG*_n_*-RM26 solution for 1 h at 37 °C. One set of dishes in each experiment was pre-saturated with 100-fold excess of unlabeled peptide, added 5 min before the addition of the radiolabeled compound. After being washed twice with serum free media, cells were detached by treatment with 0.5 mL trypsin-EDTA solution. Cell associated radioactivity was measured in the gamma-counter and presented as percentage from added radioactivity.

#### 3.3.2. Real-Time Ligand Binding Kinetics: K_D_ and B_max_ Determination

The kinetics of binding of ^111^In-NOTA-PEG*_n_*-RM26 to the PC-3 cells was measured in real-time at RT using LigandTracer instruments (Ridgeview Instruments AB, Uppsala, Sweden), as described previously [42]. In addition, binding of the most promising candidate, ^111^In-NOTA-PEG_3_-RM26, was measured using BT-474 cells. Two radioligand concentrations were used: 0.3 and 10 nM. These concentrations were chosen to receive a clear increase in signal by addition of the second concentration. Uptake was monitored for 200 min and retention for 1200 min. Interaction analysis and calculation of equilibrium dissociation constant (K_D_) was performed with TracerDrawer software (Ridgeview Instruments AB). To determine the number of binding sites per cell, B_max_, cells were incubated with 20 nM of ^111^In-NOTA-PEG_3_-RM26 at RT. When radioactivity uptake reached saturation, cells were washed twice with serum-free media and after being trypsinized, detached cells were counted and collected for radioactivity measurement. Data were used for calculation of the B_max_, on PC-3 and BT-474 cells.

#### 3.3.3. *In Vitro* Competitive Binding Assay: IC_50_ Determination

An *in vitro* competition experiment was performed on PC-3 cells using ^125^I-Tyr^4^-BBN (Perkin Elmer). The half maximal inhibitory concentration (IC_50_) was determined for non-radioactive ^nat^Ga-loaded conjugates. Cell monolayers were incubated with ^nat^Ga-NOTA-PEG*_n_*-RM26 (0–395 nM) in the presence of 0.1 pmol (~100,000 cpm) ^125^I-Tyr^4^-BBN for 3 h at 4 °C. After the incubation, the cells were collected, and cell-associated radioactivity was determined as described above. The IC_50_ values were determined using GraphPad software.

#### 3.3.4. Cellular Uptake and Internalization Assay

PC-3 cells were incubated with 2 nM of ^111^In-NOTA-PEG*_n_*-RM26 at 37 °C for 24 h. At predetermined time points, the incubation media was discarded, the cells were washed and the membrane-bound and internalized radioactivity were collected using the method described earlier [43]. In addition, the uptake and internalization of ^111^In-NOTA-PEG_3_-RM26 was studied on BT-474 cells.

### 3.4. In Vivo Studies

All animal experiments were planned and performed according to the Swedish national legislation on the protection of laboratory animals, and the study plans were approved by the local committee for animal research ethics. The biodistribution of ^68^Ga-labeled NOTA-PEG*_n_*-RM26 was evaluated in female NMRI mice (weight: 30 ± 3 g). BALB/c nu/nu female mice (weight: 19.8 ± 0.7 g) bearing PC-3 or BT-474 xenografts (10 × 10^6^ cells/mouse, implanted 3 weeks before the experiment) were used for *in vivo* binding specificity and tumor targeting study using ^68^Ga-labeled NOTA-PEG_3_-RM26. The mice for BT-474 xenografts were implanted subcutaneously with 17β-estradiol pellets (0.05 mg/21-day release, Innovative Research of America, Sarasota, FL, USA) before tumor cell inoculation and cells were inoculated in Matrigel/PBS (1:1). The average tumor size for both cell lines was 0.4 ± 0.1 g at the time of the experiment.

#### 3.4.1. Biodistribution in NMRI Mice

Female NMRI mice (3 mice per data point) were injected with 45 pmol of ^68^Ga-NOTA-PEG*_n_*-RM26 (350 or 700 kBq, 100 µL in PBS) into the tail vein. The administrated peptide dose was adjusted to 45 pmol by dilution with non-labeled NOTA-PEG*_n_*-RM26. The mice were euthanized at 1 and 2 h p.i. by intraperitoneal injection of a Ketalar-Rompun solution (10 mg/mL Ketalar and 1 mg/mL Rompun; 20 µL of solution per gram of body weight). Blood samples were collected by heart puncture. The organs of interest were collected, weighed, and their radioactivity content was measured in the gamma-counter. The organ uptake values are expressed as %ID/g except for gastrointestinal (GI) tract (with content) and carcass in which data are presented as %ID per whole sample. 

#### 3.4.2. Biodistribution and *in vivo* Binding Specificity Test in BALB/c nu/nu Mice Bearing PC-3 Prostate Cancer and BT-474 Breast Cancer Xenografts

To study tumor targeting, female BALB/c nu/nu mice bearing PC-3 and BT-474 xenografts (4 mice per data point) were intravenously injected with 45 pmol of ^68^Ga-NOTA-PEG_3_-RM26 (700 kBq, 100 µL in PBS). The mice were euthanized at 2 h p.i. and the organ radioactivity content was measured and evaluated as described above. To test the *in vivo* binding specificity, a group of animals was co-injected with 20 nmol of unlabeled peptide, and biodistribution was measured at 2 h p.i.

### 3.5. Imaging Studies

The mice bearing PC-3 or BT-474 tumors were injected with 45 pmol of ^68^Ga-NOTA-PEG_3_-RM26. The animals were sacrificed 2 h p.i. and scanned with a micro-PET/CT (Gamma Medica Inc., Salem, NH, USA). The PET and CT data were fused and analyzed using PMOD v3.13 (PMOD Technologies Ltd., Zurich, Switzerland).

### 3.6. Statistics

Statistical analyses were performed by un-paired, two-tailed t-test using Prism (version 4.00; GraphPad Software, La Jolla, CA*P* value below 0.05 was considered significant.

## 4. Conclusions

Systematic investigations of the effects of the length of PEG-spacer on *in vivo* pharmacokinetics enabled minimization of hepatic uptake. However, the influence of the length of PEG-spacer is minor when two, three, four and six ethylene glycol units are compared. 
